# TBK1 Mediates Innate Antiviral Immune Response against Duck Enteritis Virus

**DOI:** 10.3390/v14051008

**Published:** 2022-05-09

**Authors:** Dongfang Wang, Hong Huo, Gebremeskel Mamu Werid, Yassein M. Ibrahim, Lijie Tang, Yue Wang, Hongyan Chen

**Affiliations:** 1 State Key Laboratory of Veterinary Biotechnology, National Poultry Laboratory Animal Resource Center, Harbin Veterinary Research Institute, Chinese Academy of Agricultural Sciences, Harbin 150069, China; dongfangwang1990@126.com (D.W.); huohong617@163.com (H.H.); ashenafymamo@gmail.com (G.M.W.); yassin8322@gmail.com (Y.M.I.); 2 College of Veterinary Medicine, Northeast Agricultural University, Harbin 150030, China; 3 Institute of Veterinary Sciences & Pharmaceuticals, Chongqing Academy of Animal Science, Chongqing 408599, China

**Keywords:** duck enteritis virus, antiviral immunity, TBK1, type I interferon

## Abstract

Duck enteritis virus (DEV) can infect several types of waterfowl can cause high mortality and huge economic losses to the global waterfowl industry. Type I interferons (IFN) are important for host defense against virus infection through induction of antiviral effector molecules. TANK-binding kinase 1 (TBK1) is a key kinase required for the induction of type I IFNs; however, the role of TBK1 on DEV infection remains unclear. Here, we observed that the expression levels of TBK1 and IFN-β were upregulated during DEV infection in vivo and in vitro. Thus, the function of TBK1 on DEV infection was determined. The results showed that overexpression of TBK1 reduced DEV infection and knockdown of TBK1 resulted in the increased of DEV infection. Additionally, TBK1 overexpression upregulated the expression of IFN-β and a few interferon-stimulated genes (ISGs), which thus inhibited the synthesis of DEV glycoprotein B. On the other hand, the TBK1 inhibitor Amlexanox down-regulated the expression levels of IFN-β and IRF3. Interestingly, the expression levels of MAVS and GSK-3β were decreased in the cells treated with Amlexanox. Furthermore, overexpression of TBK1 activated the expression of upstream molecules MAVS and GSK-3β. Whereas, the expression of TBK1, IRF3 and IFN-β was inhibited by the GSK-3β inhibitor SB216763. Our findings suggest that DEV–stimulated TBK1 may be involved in defense against DEV infection.

## 1. Introduction

Duck viral enteritis (DVE), also known as duck plague (DP), is highly contagious and causes considerable mortality in waterfowl–domestic ducks, mallards, swans and geese of all age and species. Natural infections occur in ducklings ranging in age from seven days to mature breeders. DVE is characterized by sudden death, vascular damage and lesions in lymphoid organs, severe diarrhea and degenerative lesions in parenchymal organs [1], causing up to 100% mortality in ducklings. Currently, DEV infection has caused a huge economic loss to the global waterfowl industry. Duck enteritis virus (DEV), an enveloped double-stranded DNA virus, is the causative pathogen of DVE, and belongs to the *Herpesviridae* family [2,3,4].

The innate immune system plays an essential role in defending the host against viral infection. During viral infection, pathogen-associated molecular patterns (PAMPs) are recognized by pattern recognition receptors (PRR), which play a key role in innate immunity in the recognition of pathogens or of cellular injury. The initiation and regulation of innate immune signaling pathways are orchestrated by PPRs, including Toll-like receptors (TLRs), nucleotide-binding oligomerization domain (NOD)-like receptors (NLRs), retinoic acid-inducible gene-I (RIG-I)-like receptors (RLRs), C-type lectin receptors (CLRs), AIM2-like receptors (ALRs), and other cytosolic DNA sensors [5,6,7]. These PPRs can trigger the activation of NF-kB, type I IFN, or other inflammasome signaling pathways, leading to the production of antiviral cytokines and chemokines, which in turn establishes an antiviral state that inhibits viral replication, subsequently instructs the development of an adaptive immune response [8,9].

During DEV infection, upregulation of the expression of proinflammatory cytokines (IFN-α, IFN- β, IL-1β, IL-6) [10,11,12], RIG-I [12] and TLRs (TLR-2, 3, 21) [10] have been reported. However, the process by which innate immune response is regulated and the immune molecules involved in immune heamostais during DEV infection have not been extensively studied. The production of type I IFNs (IFN-α/β) is a fundamental host response against invading viruses [13,14,15]. TBK1 (also known as NAK or T2K), a tumor necrosis factor (TNF) receptor-associated factor NF-κB activator, is a ubiquitously expressed serine-threonine kinase, recognized for its critical role in regulating type I IFNs production [16]. Stimulation of PRRs can trigger the activation of TBK1, which activates downstream signal transduction pathways, such as multiple IFN inducing pathways, and subsequently activate the transcription factors interferon regulatory factor 3 (IRF3) or interferon regulatory factor 7 (IRF7), resulting in dimerization and IFNs production [17,18]. Type I IFN binds to IFN α/β receptor and promotes the production of numerous antiviral genes through the JAK/STAT pathway [19]. Thus, activation of TBK1 plays a crucial role in producing type I IFN as an antiviral innate immune response following infection.

Since TBK1 is a critical kinase required for the induction of type I IFNs and subsequent cellular antiviral responses, its activity must be regulated during viral infection for immune hemostasis [20]. In contrast with in mammals, the mechanisms by which TBK1 regulates virus-mediated type I IFN production in avian species remain unclear. Thus, the current study investigated the role of TBK1 in modulating host innate immune response during DEV infection in ducks. Our results showed that DEV-induced TBK1 stimulates the innate immune response, inhibiting viral replication by promoting the expression of IFN-β.

## 2. Materials and Methods

### 2.1. Cells and Viruses

DEF cells were purchased from ATCC (CCL-141, Duck Embryo Fibroblast Peking Duck; LOT:64328966, Manassas, VA, USA) and cultured in Eagle’s Minimum Essential Medium (EMEM; WISENT INC., Saint-Jean-Baptiste, QC, Canada) supplemented with 10% fetal bovine serum (FBS; Gibco, Waltham, MA, USA). The DEV CSC strain was provided by the China Institute of Veterinary Drug Control (Beijing, China) and propagated in DEF cells.

### 2.2. Viral Infection and Overexpression Assay

DEF cells were infected with DEV at a multiplicity of infection (MOI) of 0.1 or 1 for 2 h at 37 °C. Unattached viruses were removed and the cells were washed three times with phosphate buffered saline (PBS, pH 7.0). The cells were then cultured in complete medium for various time points until samples had been harvested.

Using DEF cDNA as a template, the TBK1 gene was amplified with the primers designed using primer3 [21] (Table 1) and cloned into the vector pCAGGS. The recombinant plasmid pCAGGS-TBK1 was successfully constructed. DEF cells were cultured in six-well plates. When cells reached approximately 80% confluence, they were transfected with recombinant plasmids or empty vector for 24 h. Then incubated with DEV at a MOI of 0.1 or 1 at 37 °C for 2 h and washed three times with PBS, and DEF complete medium was added. Cells were collected at 24, 36, and 48 h post-infection (hpi) for Western blot assays and Quantitative real-time PCR (qRT-PCR). Culture supernatants were collected for 50% tissue culture infective dose (TCID_50_) analysis to determine DEV replication and titers, respectively.

### 2.3. TCID_50_ Assay

TCID_50_ assays were performed on DEF cells for DEV following the Reed-Münch method. Briefly, cell monolayers in 96-well plates were inoculated with 100 µL 10-fold serial dilutions of each virus stock, with eight replicates per dilution for 2 h at 37 °C. Unattached viruses were removed and the cells were washed twice times with PBS. Then the cells were cultured in EMEM with 2% FBS for 60–72 h prior to observation of the presence of cytopathic effect.

### 2.4. RNA Interference Assay

In order to down-regulate the expression of duck TBK1, we designated the small interfering RNAs (siRNAs) targeting the duck TBK1 transcript (siTBK1) and negative control siRNAs (NC), which were synthesized by GenePharma company (Shanghai, China). All siRNAs used in this study are listed in Table 2. When cells reached approximately 60% confluence in 6-well plates, DEF cells were transfected with 200 nM siRNA or siNC with X-tremeGENE siRNA transfection reagent (Roche, Indianapolis, IN, USA) according to the manufacturer’s instructions. After 24 h of transfection, the efficiency of RNA silencing was verified by qRT-PCR. Furthermore, at 24 h after transfection, DEF cells were infected with DEV at MOI of 0.1 at 37 °C for 2 h, and washed twice with PBS. Subsequently, the cell medium was replaced with EMEM containing 2% FBS and incubated at 37 °C. Cells were then collected at 48 hpi for TCID_50_ and qRT-PCR analysis. The supernatants were collected for the detection of viral DNA copies and viral titers, respectively.

### 2.5. RNA Extraction and qRT-PCR

Total RNA was extracted from DEF cells and different tissues of DEV ducks by using an RNA Extraction Kit (Axygen, Union City, CA, USA) according to the manufacturer’s instructions. One µg RNA samples were reverse transcribed into cDNA by using a PrimeScript™RT Reagent Kit with gDNA Eraser (Takara, Japan). For the detection of the expression of target genes, specific primers were designed using primer3 [21] (Table 3). qRT-PCR reaction was performed with 2× SYBR Green qPCR Master Mix (Rox) (Takara, Japan). RT-PCR was performed under the following conditions: 95 °C for 30 s for initial denaturation, followed by 40 cycles of denaturation for 5 s at 95 °C, 55 °C for 30 s, 72 °C for 30 s. The expression levels of each gene were normalized to the expression level of *GAPDH*, and the 2^−ΔΔCt^ method was used to analyze the relative level of gene expression. All standards, controls and infected samples were tested in triplicate on the same plate.

Viral DNA was extracted from the tissues and infected DEF cells using a Viral DNA Extraction Kit (Omega, Norcross, GA, USA). Viral DNA was quantified by qRT-PCR with Premix Ex Taq (probe qPCR) (TaKaRa, Japan) using published primers [12]. TaqMan qRT-PCR was performed under the following conditions: 95 °C for 30 s for initial denaturation, followed by 40 cycles at 95 °C, 60 °C for 50 s. DEV copies were quantified by using the standard curve method established in our laboratory for absolute quantitative analysis. The primers used in qRT-PCR were listed in Table 3.

### 2.6. Western Blot Assay

Treated cells were lysed by RIPA Lysis Buffer containing nuclease and protease inhibitors at 4 °C for 30 min. Cell lysates were collected into 1.5 mL tubes, centrifuged at 12,000× *g* for 5 min, and boiled at 100 °C with loading buffer for 10 min. Samples were separated by SDS-PAGE under reducing conditions and transferred to a polyvinylidene difluoride (PVDF) membrane (Merck Millipore, Temecula, CA, USA). Membranes were blocked in 5% skim milk in PBST buffer for 2 h at room temperature and then incubated with a primary antibody for 60 min. A mouse-derived monoclonal antibody against glycoprotein B (gB) was previously developed by our laboratory. The mouse anti-gB mAb as a primary antibody. The mouse anti-β-actin antibody (Sigma, Northbrook, IL, USA) diluted at 1:10,000 was used as primary antibody. After three times washing with PBST, the membranes were incubated with IRDye-800-CW-conjugated goat anti-mouse IgG antibody (LI-COR Biosciences, Lincoln, NE, USA), diluted with 1:10,000 for 1 h at room temperature. Membranes were washed as described above. Finally, the membranes were visualized using an Odyssey Infrared Fluorescence Scanning Imaging System (Li-Cor Biosciences, USA).

### 2.7. Animal Infection Experiments

Prior to performing experiments, the use of animals for the experiment was approved by the Animal Ethics Committee of Harbin Veterinary Research Institute on December 2017 (Animal Ethics Committee approval number: SYXK (Hei) 2017-009). All animal experiments were performed according to the guidelines for Animal Experimentation of the Harbin Veterinary Research Institute. Eighteen 21-day-old SPF ducks were obtained from the Harbin Veterinary Research Institute. Nine ducks were intramuscularly inoculated with 0.2 mL of DEV per duck (2 × 10^4^ TCID_50_/mL) as the infection group, while the other nine ducks received 0.2 mL of DMEM per duck as the mock control group. Three ducks in each group were killed at 1, 3 and 5 days post-infection (dpi), respectively. Tissue samples including liver, brain, lung, kidney, spleen, bursa, thymus, small intestine and large intestine were collected for further analysis.

### 2.8. Drug Treatments

For the inhibition experiments, DEF cells were pretreated with the TBK1 inhibitor Amlexanox (40 µM; MCE, South Brunswick, NJ, USA), the GSK-3β inhibitor SB216763 (60 µM; MCE, USA) or equal amount of DMSO (carrier control) for 24 h prior to viral infection. At 24 hpi, cells were collected for subsequent analysis.

### 2.9. Statistical Analysis

All experiments were carried out in triplicates and statistical analyses were performed using Student’s *t*-test on GraphPad Prism 5 software (GraphPad Software Inc., San Diego, CA, USA). Data were reported as means ±standard deviations (SD). *p* < 0.05 was considered statistically significant, *p* < 0.01 was considered highly significant, and *p* < 0.001 was considered extremely significant.

## 3. Results

### 3.1. DEV Infection Results in the Increase of TBK1 and IFN-β Expression In Vitro

**Figure 1:**
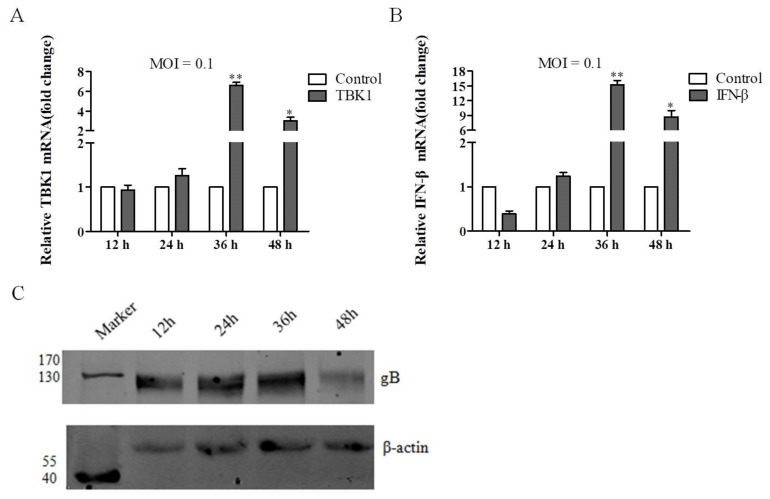
Effects of DEV infection on the expression levels of TBK1 and IFN-β in vitro. DEF cells were infected with DEV at a MOI of 0.1 for indicated time points, and cell samples were collected, respectively. (**A**) The qRT-PCR results of TBK1 gene expression in DEF cells at a MOI of 0.1. (**B**) The qRT-PCR results of IFN-β gene expression in DEF cells at a MOI of 0.1. (**C**) The expression levels of DEV gB in DEF cells at indicated time points by western blot assays, with β-actin protein as the control. Mean ± SD of three technical replicates are shown. * *p* < 0.05, ** *p* < 0.01.

To determine if DEV infection can activate the innate immune response in vitro, we infected DEF cells with DEV at a MOI of 0.1, and cells and supernatants were collected at indicated time points. The qRT-PCR results showed that the expression level of TBK1 was increased and reached peak at 36 hpi (Figure 1A). Meanwhile, IFN-β mRNA levels showed a similar trend to TBK1 mRNA levels (Figure 1B). These results revealed that TBK1 and IFN-β were significantly induced upon DEV infection in vitro, indicating that TBK1 and IFN-β might play important roles during DEV infection. In addition, western blot results showed that the expression levels of DEV gB were increased with time, indicating virus infection was successful (Figure 1C).

### 3.2. DEV Infection Induces TBK1 and IFN-β Expression In Vivo

**Figure 2:**
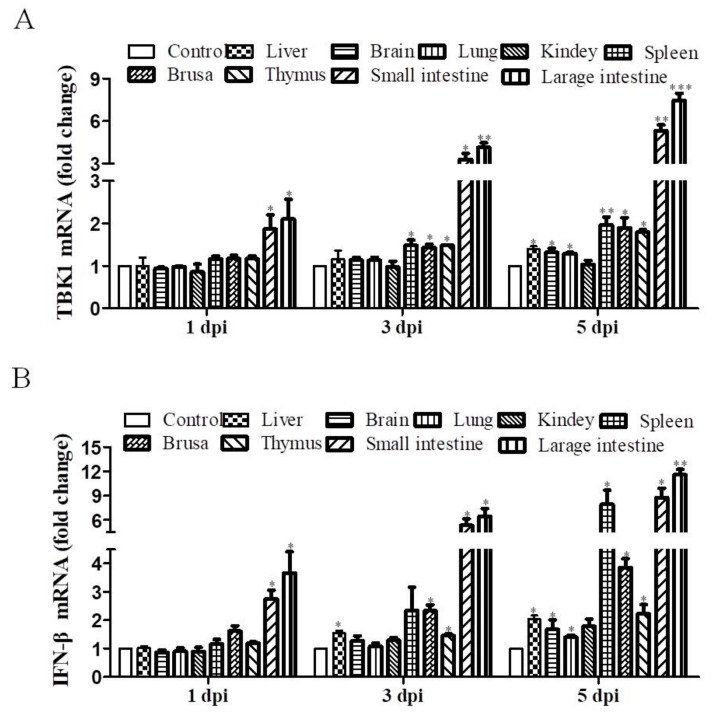
DEV infection induces TBK1 and IFN-β expression in vivo. (**A**,**B**) Eighteen 21-day-old SPF ducks were divided into an infection group randomly (intramuscular injection of 0.2 mL DEV (2 × 10^4^ TCID_50_) and a control group (intramuscular injection of 0.2 mL EMEM). For qRT-PCR analysis, three ducks from each group were killed and tissue samples were collected at 1, 3, and 5 dpi. (**A**) TBK1 mRNA expression levels in different organs and at the indicated time points. (**B**) IFN-β mRNA expression levels in different organs and at the indicated time points. Mean ± SD of three technical replicates are shown. * *p* < 0.05, ** *p* < 0.01, *** *p* < 0.001.

To determine whether DEV infection can activate the host innate immune response in vivo, animal infection experiments were carried out, we collected tissue samples of DEV infected ducks. The qRT-PCR results showed that TBK1 expression was upregulated in most tissues of DEV infected ducks, such that TBK1 expression was increased in the small intestine and large intestine with time at 1, 3, and 5 dpi; slight upregulation of TBK1 expression was observed in spleen, brusa, and thymus at 3 and 5 dpi (Figure 2A). These data suggest that DEV infection could rapidly activate the antiviral response in DEV-targeted tissues of ducks, indicating the involvement of TBK1 during DEV infection in vivo. Therefore, we assessed IFN-β expression in different tissues as well. The qRT-PCR results showed that the expression of IFN-β had similar trends to TBK1 at the indicated time points (Figure 2B). Specifically, the IFN-β expression was significantly increased in the digestive tract as well as immune organs. These data suggest that DEV infection does not induce immunosuppression in ducks, but can rapidly activate innate immunity to fight against DEV infection. Overall, in vitro and in vivo results indicate the role of TBK1 for the induction of type I IFNs and subsequent cellular antiviral responses.

### 3.3. Overexpression of TBK1 Inhibits DEV Infection

**Figure 3:**
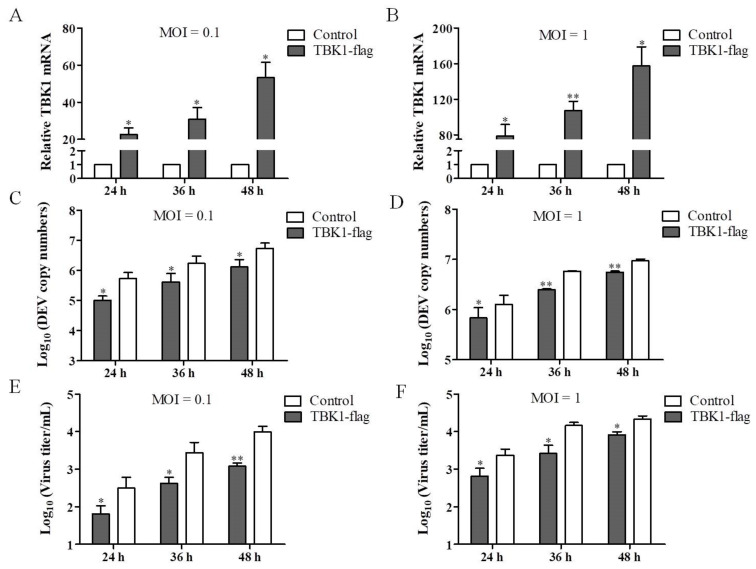
Overexpression of TBK1 inhibits DEV infection. (**A**–**E**) DEF cells were transfected with pCAGGS-TBK1 and infected with DEV at a MOI of 0.1 or 1, for 24 h, 36 h, 48 h, respectively. (**A**) The qRT-PCR results of TBK1 expression at a MOI of 0.1. (**B**) The qRT-PCR results of TBK1 expression at a MOI of 1. (**C**) DEV copy number determined by qRT-PCR at a MOI of 0.1. (**D**) DEV copy number determined by qRT-PCR at a MOI of 1. (**E**) DEV titer in the TCID_50_ assay titer at a MOI of 0.1. (**F**) DEV titer in the TCID_50_ assay titer at a MOI of 1. Mean ± SD of three technical replicates are shown. * *p* < 0.05, ** *p* < 0.01.

**Figure 4:**
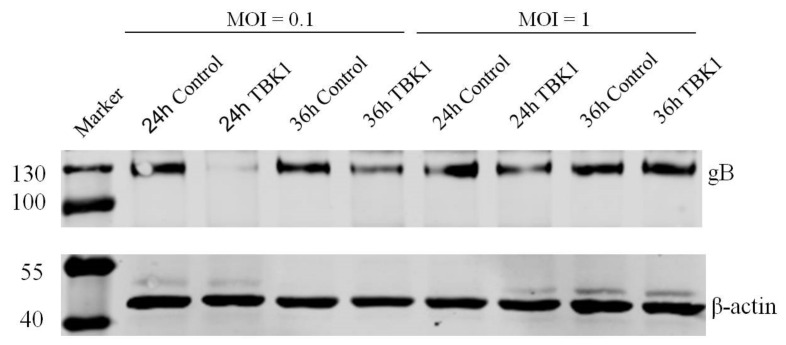
Overexpression of TBK1 decreased gB protein expression during DEV infection. DEF cells were transfected with pCAGGS-TBK1 and infected with DEV at a MOI of 0.1 or 1 for 24 h, 36 h by western blot analysis. Expression of gB protein in the TBK1 overexpressed group compared with the control group were analyzed by western blot assays, with β-actin protein as the control.

To determine whether TBK1 plays a role in DEV infection, DEF cells were transfected with pCAGGS-TBK1, and then infected with DEV at a MOI of 0.1 or 1, for 24, 36, and 48 h, respectively. The qRT-PCR results showed that overexpression of TBK1 increased the TBK1 expression at different MOIs over time (Figure 3A,B). Compared with the control group, the TBK1 overexpression resulted in the decrease of DEV copy number (Figure 3C,D) and DEV titer in the TCID_50_ assay (Figure 3E,F) over time. Furthermore, DEF cells were transfected with pCAGGS-TBK1 and infected with DEV. Western blot result showed that the gB protein expression were reduced in TBK1-transfected cells (Figure 4), indicating that overexpression of TBK1 inhibits DEV infection. Overall, these data support the key role of TBK1 in DEV infection.

### 3.4. Knockdown of Endogenous TBK1 Expression Promotes DEV Infection

**Figure 5:**
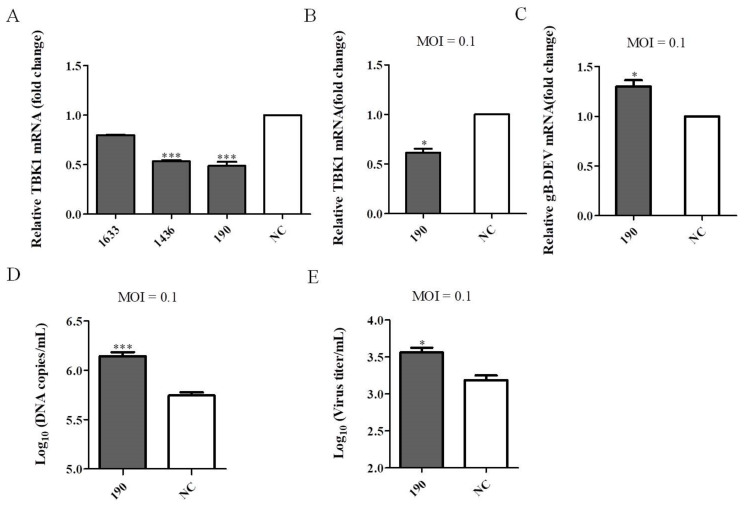
Knockdown of TBK1 promotes DEV infection. (**A**) TBK1 was knocked down in DEF cells using siRNA 24 h prior to DEV infection. The knockdown efficiencies of siRNAs were evaluated by qRT-PCR. (**B**–**E**) DEF cells were transfected with siTBK1 or siNC, and then infected with DEV at an MOI of 0.1, respectively. (**B**) One specific siRNA (siTBK1-190) to confirms the TBK1 expression with DEV at a MOI of 0.1 by qRT-PCR. (**C**) The expression of DEV gB mRNA level in siTBK1-transfected DEF cells infected with DEV at a MOI of 0.1 by qRT-PCR. (**D**) The DEV copy number by qRT-PCR. (**E**) The DEV titer in the TCID_50_ assay. Mean ± SD of three technical replicates are shown. * *p* < 0.05, *** *p* < 0.001.

To further investigate the effect of TBK1 on DEV infection, TBK1 was knocked down in DEF cells 24 h prior to DEV infection using TBK1 siRNA. The knockdown efficiency of siRNAs was confirmed by qRT-PCR, and siTBK1-190 showed the best effect (Figure 5A). Therefore, siTBK1-190 was selected for the next experiment. As shown in Figure 5B, we confirmed the effects of siTBK1-190 on TBK1 expression in DEV-infected DEF cells (Figure 5B). The expression of DEV gB mRNA level was significantly upregulated in siTBK1-transfected DEF cells (Figure 5C). Similarly, both the viral genome copy number and viral titer were significantly increased in TBK1 specific siRNA treated cells, as determined by qRT-PCR and TCID_50_ analysis, respectively (Figure 5D,E). Collectively, these results demonstrate that knockdown of TBK1 expression significantly promotes DEV infection.

### 3.5. Overexpression of TBK1 Induces IFN-β and ISGs Expression

**Figure 6:**
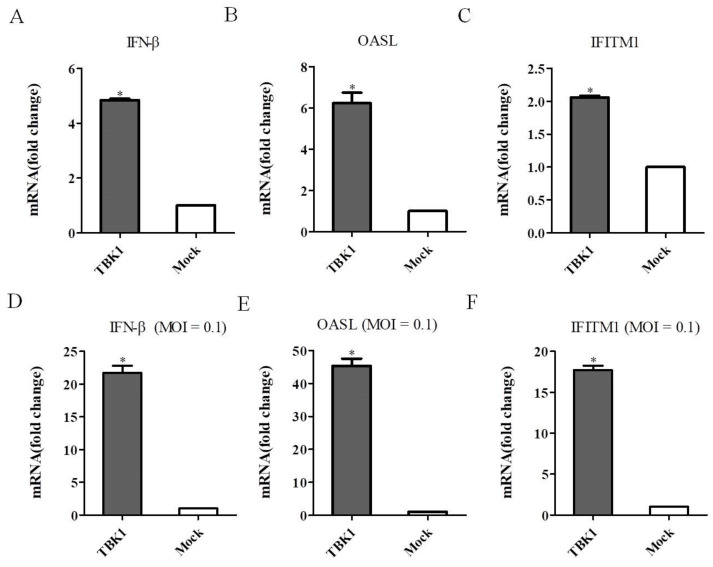
IFN-β and ISGs expression levels in DEF cells. (**A**–**C**) DEF cells were transfected with pCAGGS-TBK1 expressing TBK1 for 48 h and IFN-β, OASL and IFITM1 expression levels were detected by qRT-PCR. (**D**–**F**) DEF cells were transfected with pCAGGS-TBK1 expressing TBK1 for 24 h, and then infected with DEV at a MOI of 0.1. DEF cells were then collected and IFN-β, OASL and IFITM1 expression levels were detected by qRT-PCR. Significant differences in IFN-β, OASL and IFITM1 expression levels between TBK1-transfected and mock-transfected DEF cells were assessed using the Student’s t-test. Mean ± SD of three technical replicates are shown. * *p* < 0.05.

To determine whether TBK1 regulates viral replication through regulating type I IFN signaling pathway, DEF cells were transfected with pCAGGS-TBK1, and then infected with DEV. The qRT-PCR results showed that compared with the mock control, overexpression of TBK1 significantly upregulated IFN-β expression (Figure 6A,D). We further examined the effect of TBK1 on type I IFN signaling molecules by analyzing the expression levels of OASL and IFITM1 by qRT-PCR. As shown in Figure 6B,E, overexpression of TBK1 upregulated OASL expression in either uninfected or infected DEF cells. Overexpression of TBK1 upregulated IFITM1 expression in either uninfected or infected DEF cells as well (Figure 6C,F). These results indicated that overexpression of TBK1 induced the expression of IFN-β and ISGs, thereby inhibited DEV infection.

### 3.6. Overexpression of TBK1 Activates MAVS and GSK-3β in DEF Cells

**Figure 7:**
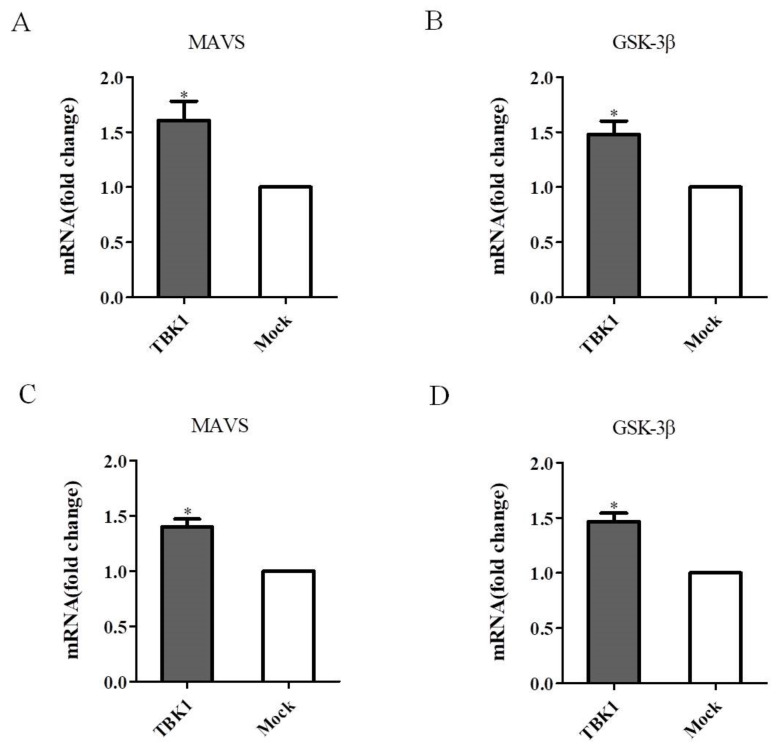
MAVS and GSK-3β expression levels in DEF cells. (**A**,**B**) DEF cells were transfected with pCAGGS-TBK1 expressing TBK1 for 48 h and MAVS and GSK-3β expression levels were detected by qRT-PCR. (**C**,**D**) DEF cells were transfected with pCAGGS-TBK1 expressing TBK1 for 24 h, and then infected with DEV at a MOI of 0.1. DEF cells were then collected and MAVS and GSK-3β expression levels were detected by qRT-PCR. Significant differences in MAVS and GSK-3β expression levels between TBK1-transfected and mock-transfected DEF cells were assessed using the Student’s *t*-test. Mean ± SD of three technical replicates are shown. * *p* < 0.05.

DEF cells were transfected with pCAGGS-TBK1, and then infected with DEV. The qRT-PCR results showed that compared with the control group, overexpression of TBK1 significantly upregulated MAVS (mitochondrial antiviral-signaling protein) expression in either uninfected or infected DEF cells (Figure 7A,C), implying that overexpression of TBK1 could activate MAVS antiviral signal transduction. In addition, the qRT-PCR results showed that overexpression of TBK1 significantly upregulated GSK-3β (Glycogen synthase kinase 3β) expression in either uninfected or infected DEF cells (Figure 7B,D), implying that overexpression of TBK1 could activate the upstream molecule GSK-3β.

### 3.7. Inhibition of TBK1 Decreases the Antiviral Signaling

**Figure 8:**
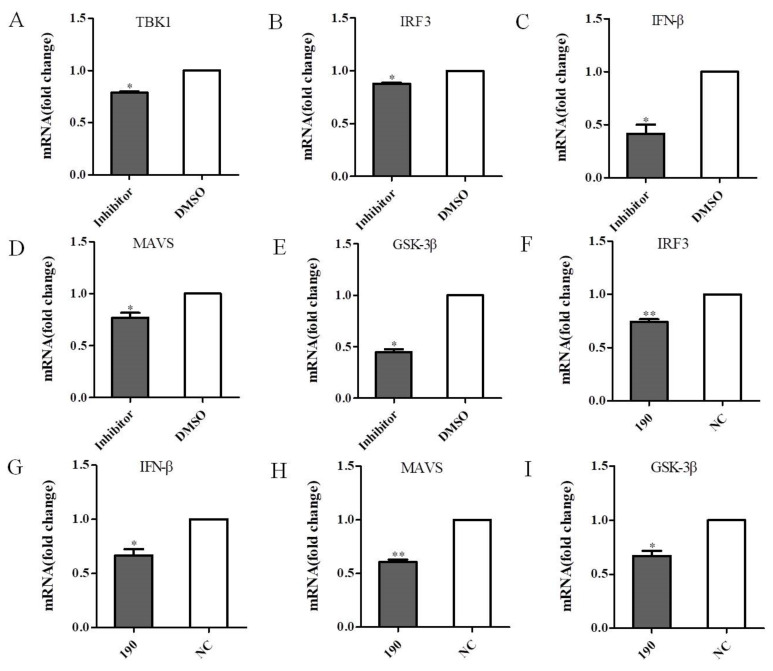
Inhibition of TBK1 decreases the antiviral signaling. (**A**–**E**) DEF cells were treated with 40 µM Amlexanox as the experimental group, and DEF cells were treated with DMSO as the carrier control, and then infected with DEV at a MOI of 0.1 prior to qRT-PCR analysis. (**A**) TBK1 mRNA expression levels. (**B**) IRF3 mRNA expression levels. (**C**) IFN-β mRNA expression levels. (**D**) MAVS mRNA expression levels. (**E**) GSK-3β mRNA expression levels. (**F**–**H**) DEF cells transfected with siTBK1-190 and infected with DEV at a MOI of 0.1. (**F**) IRF3 mRNA expression levels. (**G**) IFN-β mRNA expression levels. (**H**) MAVS mRNA expression levels. (**I**) GSK-3β mRNA expression levels. Mean ± SD of three technical replicates are shown. * *p* < 0.05, ** *p* < 0.01.

To further investigate whether the inhibitor of TBK1 have the same effect as siRNA, DEF cells were treated with the TBK1 inhibitor Amlexanox or carrier control (DMSO) for 24 h and then infected with DEV. The qRT-PCR results revealed that Amlexanox was able to down-regulate TBK1 expression (Figure 8A), and the expression levels of IRF3 was decreased as well (Figure 8B). Moreover, the qRT-PCR results revealed that Amlexanox was able to down-regulate IFN-β expression (Figure 8C). Interestingly, the expression levels of MAVS and GSK-3β were also decreased in Amlexanox-treated cells (Figure 8D,E). On the other hand, DEF cells transfected with siTBK1-190 and infected with DEV. The qRT-PCR results showed that the expression levels of IRF3 and IFN-β were decreased in siTBK1-transfected DEF cells (Figure 8F,G). In addition, siTBK1-transfected DEF cells resulted in the decrease of the expression of MAVS and GSK-3β as determined by qRT-PCR (Figure 8H,I). Taken together, these data suggest that the TBK1 inhibitor Amlexanox has similar effect to the TBK1 siRNA in antiviral signaling, further supporting the key role of TBK1 in DEV infection.

### 3.8. Effect of GSK-3β-Associated Signaling on TBK1 in Antiviral Signaling

**Figure 9:**
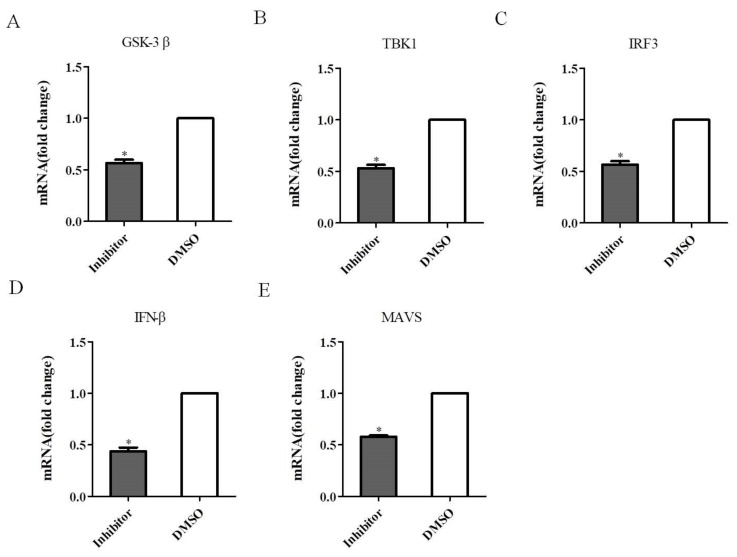
Effect of GSK-3β-associated signaling on TBK1 in antiviral signaling. For qRT-PCR analysis, DEF cells were treated with 60 µM GSK-3β inhibitor for 24 h and infected with DEV at a MOI of 0.1 as an experimental group and treated with DMSO as the carrier control. (**A**) GSK-3β mRNA expression levels. (**B**) TBK1 mRNA expression levels. (**C**) IRF3 mRNA expression levels. (**D**) IFN-β mRNA expression levels. (**E**) MAVS mRNA expression levels. Mean ± SD of three technical replicates are shown. * *p* < 0.05.

Recent studies showed that GSK-3β exerts its innate antiviral property by regulating the phosphorylation of TBK1 and interacting with other signaling molecules [22]. To explore the effect of GSK-3β-associated signaling on TBK1, DEF cells were treated with the GSK-3β inhibitor SB216763 or carrier control (DMSO) for 24 h and then infected with DEV. The qRT-PCR results showed that the GSK-3β inhibitor SB216763 successfully down-regulated the expression of GSK-3β (Figure 9A), and that GSK-3β inhibitor SB216763 was able to down-regulate TBK1 expression (Figure 9B). Subsequently the expression levels of IRF3 were decreased as detected by qRT-PCR (Figure 9C). Moreover, the qRT-PCR results showed that SB216763 down-regulated IFN-β expression (Figure 9D). Interestingly, the expression levels of MAVS were decreased as well (Figure 9E). These findings indicate an association of GSK-3β with TBK1 in antiviral signaling.

## 4. Discussion

Viral DNA in the cytosol induces type I IFNs via the endoplasmic reticulum membrane protein STING, activating the transcription factors NF-kB and IRF3 or IRF7 via the kinases IKK and TBK1 [17,18]. TBK1 is a kinase and ubiquitin-like domain (ULD)-containing protein required to induce type I IFNs and subsequent cellular antiviral responses [13,15,16]. While the kinase domain helps phosphorylation of substrates, such as IRF3 [23], the ULD domain regulates kinase activation and interactions with other proteins in the TBK1 pathway [24]. Optimal activation of TBK1 is crucial for initiating the innate antiviral response and maintaining immune homeostasis. However, the mechanism by which TBK1 regulates antiviral host immune response, especially during DEV infection, has not been fully elucidated. In this study, we demonstrated the role of TBK1 in the innate immune response during DEV infection in ducks.

To confirm the role of TBK1 in DEV replication, TBK1 was overexpressed in DEF cells and then cells were infected with DEV. Overexpression of TBK1 increased TBK1 mRNA expression levels while decreasing viral copy number and viral titer over time. Furthermore, During TBK1 overexpression, the mRNA expression levels of IFN-β and ISGs molecule– OASL and IFITM1, were all upregulated in DEF cells. Similarly, Hua, et al. [25] reported that TBK1 overexpression induced IFN-β production through the activation of IRF1 and NF-κB in DEV infected DEF cells. Moreover, in the current study, western blot results showed that expression of gB protein which is essential for attachment and penetration of free virions [26], cell fusion and infection transmission [27,28] was significantly decreased in TBK1 overexpressed cells, implying the role of TBK1 during DEV infection. Whereas TBK1 knockdown by siRNA resulted in an increased DEV copy number, DEV titer and gB protein expression, further supporting the role of TBK1 in DEV infection. Previous reports also showed a significant reduction in poly(I:C) or Sendai virus-inducted IFN-β expression following TBK1 siRNA knockdown [25]. Moreover, significantly reduced mRNA expression levels of IRF3 and IFN-β were found in TBK1 knockdown chicken embryonic fibroblast cells (CEF) following avian leukosis (ALV) infection [29]. This highlights the role of TBK1 in interferon pathway regulation during viral infection in ducks and chickens.

During DEV infection in vitro, the mRNA expression levels of TBK1, IFN-β and DEV gB showed similar expression pattern across different time points. Similarly, an increased expression of TBK1, IRF3 and IFNβ was reported in chicken fibroblasts following ALV infection [29]. Similar to the in vitro results, TBK1 and IFN-β were highly expressed in DEV-infected ducks. In response to DEV infection, compared to the control group, TBK1 and IFN-β expression were significantly increased in the target organs–digestive tract at all time points, which were increased in the immune organs at 3 dpi and 5 dpi. Highest DEV viral load was previously reported in duckling intestine and spleen [12], further indicating a direct correlation between viral load and TBK1 and IFN-β induction. Similarly, in chickens, following ALV infection, TBK1 was found upregulated in spleen, bursa of Fabricius and thymus [29]. Though TBK1 expression following DEV infection in ducks have not been reported yet, in healthy ducks, TBK1 expression was found higher in the liver, heart, and duodenum, but lower in the lung, spleen, thymus, and bursa of Fabricius [25]. Contrary to the lower levels of TBK1 expression in liver, higher expression in spleen, lung, thymus and bursa of Fabricius has been reported in healthy chickens [29]. Such differences in tissue-based expression of TBK1 in healthy ducks and chickens might arise from variations in the environment and/or species.

MAVS and TBK1 enhancer GSK-3β play major role in regulating TBK1/IRF3 dependent antiviral responses in mammals [30,31,32]. In the current study, we have demonstrated the link between TBK1 inhibitors and IFN-β responses. Amlexanox, a known TBK1 inhibitor in mammalian cells [33], was able to down-regulate the mRNA expression levels of TBK1, IRF3, IFN-β. Interestingly, the expression levels of MAVS and GSK-3β also were decreased. Moreover, overexpression of TBK1 activates MAVS antiviral signal transduction and GSK-3β in DEF cells. These results further support the role of TBK1 in the immune response of avian species.

Recent studies showed that GSK-3β exerts its innate antiviral property by regulating the phosphorylation activity of TBK1 and interacting with various signaling molecules [22,34]. GSK-3β promotes TBK1 self-aggregation and autophosphorylation at Ser172, both of which are for virus-induced IRF3 activation and IFN-β induction [32]. In this study, a GSK-3β inhibitor, SB216763 [35], was found to successfully suppress the expression of GSK-3β, TBK1, IRF3, IFN-β. Interestingly, the expression levels of MAVS and GSK-3β also were decreased. Taken together, the results of TBK1 overexpression, TBK1 knockdown, in vitro and in vivo experiments support the role of TBK1 in IFN-β induction in ducks following viral infection.

IRF3, which is activated by TBK1, is thought to be a result of STING activation, which drives immune responses against DNA viruses and tumors [36,37]. In response to viral infection or nucleic acid exposure, IRF3 hyperphosphorylate, dimerize and translocate to the nucleus as an active transcription factor [37]. The downregulation of IRF3 by TBK1 and GSK-3β inhibitors found in this study adds to the evidence that TBK1 plays a role in IFN-β regulation during DEV infection in ducks.

## 5. Conclusions

In this study, DEV infection induced TBK1 expression both in vitro and in vivo. The up-regulated TBK1 can inhibit DEV infection through inducing the antiviral IFN-β production and ISGs expression. Our findings reveal that TBK1 plays an important role in resistance to DEV infection and host antiviral responses. Furthermore, the findings of this study provide new insights into our understanding of the viral replication process, host antiviral immunity, pathogenicity, disease prevention and may contribute to the development of new antiviral drugs. However, further studies are required to elucidate the precise mechanisms underlying the antiviral activity of TBK1 in DEV replication.

## Figures and Tables

**Table 1:** Primer sequence for TBK1 gene amplification.

Primer Name	Sequence (5′-3′)	Accession Number	Target Region (bp)
TBK1-F	CAGACCATCGATATGCAGGACTTCAAATTA	XM_027456350.2	191–2375
TBK1-R	ATTTGCGGCCGCGATACAGTCCACATTCC		

**Table 2:** List of siRNAs used to knockdown TBK1.

siRNAs		Sense (5′–3′)	Antisense (5′–3′)
siTBK1	TBK1-190	GCUGUUGUCUGACAUUCUATT	UAGAAUGUCAGACAACAGCTT
	TBK1-1436	GCCUGUAGAGUUGCCAGUUTT	AACUGGCAACUCUACAGGCTT
	TBK1-1633	GGAAUCAUCAGAAGUGGAUTT	AUCCACUUCUGAUGAUUCCTT
siNC	negative control	UUCUCCGAACGUGUCACGUTT	ACGUGACACGUUCGGAGAATT

**Table 3:** Primer sequences used for qRT-PCR.

Primer Name	Sequence (5′-3′)	Accession Number	Target Region (bp)
TBK1-qF	ACTGCTCACACCTGTCCTTG	KY963947.1	825–925
TBK1-qR	TTCGGTGCAGGATGTCACTT		
IFN-β-qF	AGATGGCTCCCAGCTCTACA	NM_001310827.2	213–422
IFN-β-qR	AGTGGTTGAGCTGGTTGAGG		
OASL-qF	AAGAAGATCAACAGCCGCCA	KU569293.1	1398–1508
OASL-qR	CTTGGGCTGGACGTTGTAGT		
IFITM1-qF	TCAAGGCCCGAGATAGGACA	KX811738.1	233–347
IFITM1-qR	ATGATGGTGCCCACGATACC		
GAPDH-qF	GCCTCTTGCACCACCAACT	AY436595.1	344–436
GAPDH-qR	GGCATGGACAGTGGTCATAAGAC		
DEV-qF	TGGGAAGGCTTTCGGTCGC	Reference [12]	
DEV-qR	CATTCGCGCCTTTGCTAAATTCTCT		

## Data Availability

Not applicable.
